# Percutaneous Coronary Intervention versus Coronary Artery Bypass Graft in Acute Coronary Syndrome patients with Renal Dysfunction

**DOI:** 10.1038/s41598-018-20651-3

**Published:** 2018-02-02

**Authors:** Xiaojia Zhang, Liangping Hu, Wen Zheng

**Affiliations:** 1 Consulting Center of Biomedical Statistics, Academy of Military Medical Sciences, Beijing, 100850 China; 2 Specialty Committee of Clinical Scientific Research Statistics of World Federation of Chinese Medicine Societies, Beijing, 100029 China; 3 Beijing Anzhen Hospital, Capital Medical University, Beijing, 100029 China

## Abstract

ACS patients with renal dysfunction tend to have a poorer prognosis than those with normal renal function. This retrospective cohort study was performed using The Second Drug-Eluting Stent Impact on Revascularization Registry, a retrospective registry, to evaluate the time-dependent relative risk of revascularization strategies in ACS patients with renal dysfunction. The study demonstrated that the short-term MACCE rate was lower after PCI than CABG. However, there was no significant difference in long-term MACCE rate. Subgroup analyses based on the degree of renal dysfunction resulted in similar findings. The revascularization strategy was identified as a time-dependent covariate by the time-dependent Cox model, and the regression coefficient was ‘−1.124 + 0.344 × ln (time + 1)’. For the entire object group and the separate subgroups, PCI was initially associated with a lower hazard for MACCE than CABG after revascularization, then the hazard ratio increases with time. In conclusion, the hazard ratio for MACCE in PCI relative to CABG is time-dependent. PCI tends to have a lower risk for MACCE than CABG in the short-term, then the hazard ratio increases with time.

## Introduction

Cardiovascular disease is the leading cause of morbidity and mortality in patients with renal dysfunction^1^. Acute coronary syndrome (ACS) are severe types of cardiovascular disease, and affect millions of people globally each year. ACS patients with renal dysfunction have a poorer prognosis than those with normal renal function. Impaired renal function was identified as a high-risk independent factor for subsequent cardiovascular events^2,3^. Additionally, patients with renal dysfunction tend to have a higher prevalence of diabetes mellitus and hyperlipidemia. These typical ACS risk factors facilitate the pathogenesis of atherosclerosis in most patients^4^.

Percutaneous coronary intervention (PCI) and coronary artery bypass graft (CABG) are alternative revascularization strategies for ACS patients. However, the treatment of ACS patients with renal dysfunction is challenging, due to higher underlying comorbidities^5^, increased thrombotic and bleeding risk^6–8^, and coronary lesion calcification and complexity^6,9^. A few meta-analyses have assessed revascularization strategies for patients with renal dysfunction, but they have shown inconsistent findings, because most studies that were used were retrospective studies and their heterogeneity level was high^10–13^. There is a lack of RCTs assessing revascularization strategies for ACS patients with renal dysfunction. A number of randomized controlled trials (RCTs) have evaluated the safety and efficacy of PCI versus CABG for ACS patients, but they have excluded patients with impaired renal function^14–16^. Most of the studies apply typical Cox model to evaluate the relative risk of revascularization strategies for major adverse cardiac and cerebrovascular events in the short term and long term. Consequently, these studies ignored the time-dependent associations in revascularization strategies with their outcomes. This study aims to evaluate the fluctuant relative risk of revascularization strategies for ACS patients with renal dysfunction, for the purpose of searching for optimal revascularization strategy in different situations.

## Methods

### Study population

The Second Drug-Eluting Stent Impact on Revascularization Registry (DESIRE-2) was a retrospective registry study, which enrolled 6005 coronary artery disease patients who underwent revascularization between July 2003 and October 2005 at Beijing Anzhen Hospital. The study protocol conforms to the ethical guidelines of the 1975 Declaration of Helsinki as reflected in a priori approval by the institutional review board of Beijing Anzhen Hospital. Due to retrospective enrollment, written informed consent from the patients was waived. In the current study, the inclusion criteria were the following: (1) patients with renal dysfunction defined as those with an estimated glomerular filtration rate (eGFR) of less than 90 ml/(min · 1.73 m^2^); (2) patients with ACS defined as those diagnosed with ST-elevated MI, non-ST-elevated MI or unstable angina. Unstable angina was defined as angina pectoris or equivalent ischemic discomfort with at least one of three features: (a) it was severe and of new onset (i.e. within the 4–6 weeks prior); (b) it occurred in a progressive pattern; (c) it occurred at rest (or with minimal exertion), usually lasting >10 min. (3) patients with multivessel disease and those with single vessel disease; (4) patients undergoing either PCI or CABG procedure. The patients who underwent unsuccessful revascularization procedures or those with missing renal function measures were excluded from the study. A successful PCI procedure was defined as a residual stenosis of less than 20% and forward flow TIMI 3 after surgery. A successful CABG procedure was defined as the patency in the grafts after surgery.

### Data collection

Desire-2 recorded baseline demographics, clinical presentations, laboratory variables, procedural variables and outcome data. The variables recorded included gender, age, body mass index (BMI), body surface area, diabetes mellitus, hypertension, history of cerebrovascular disease, old myocardial infarction (OMI), smoking history, peripheral vascular disease (PVD), chronic obstructive pulmonary disease (COPD), acute coronary syndrome, systolic blood pressure, diastolic blood pressure, mean arterial pressure, atrial fibrillation and atrial flutter, left ventricular ejection fraction (LVEF), hemoglobin, white blood cell (WBC), neutrophils, blood urea nitrogen (BUN), serum creatinine (CR), total cholesterol (TC), triglyceride (TG), low density lipoprotein cholesterol (LDL-C), high density lipoprotein cholesterol (HDL-C), fasting blood glucose (FBG), time from admission to revascularization, vessel involvement, left main disease, revascularization strategy, complete revascularization, ostial lesions, left anterior descending disease, and use of statin, beta blocker, Calcium channel blocker, nitrate, ACE-I and ARB.

The estimated glomerular filtration rate was calculated using the Modification of Diet in Renal Disease (MDRD) study formula as follows^17^:

1 $$\begin{array}{ccc}{\rm{G}}{\rm{F}}{\rm{R}} & = & 186.3\times {({\rm{s}}{\rm{e}}{\rm{r}}{\rm{u}}{\rm{m}}{\rm{c}}{\rm{r}}{\rm{e}}{\rm{a}}{\rm{t}}{\rm{i}}{\rm{n}}{\rm{i}}{\rm{n}}{\rm{e}}{\rm{m}}{\rm{g}}/{\rm{d}}{\rm{l}})}^{-1.154}\times ag{e}^{-0.203}\\  &  & \times \,0.742({\rm{i}}{\rm{f}}\,{\rm{f}}{\rm{e}}{\rm{m}}{\rm{a}}{\rm{l}}{\rm{e}})\times 1.212({\rm{i}}{\rm{f}}\,{\rm{b}}{\rm{l}}{\rm{a}}{\rm{c}}{\rm{k}})\end{array}$$

### Outcome data

The outcomes were major adverse cardiac and cerebrovascular events (MACCE), which included all-cause mortality, repeat revascularization, non-fatal myocardial infarction and ischemic stroke. Repeat revascularization was defined as target or non-target vessel revascularization after the index procedure. Myocardial infarction was defined as repeat ischemic chest pain, electrocardiographic dynamic changes and elevated levels of the myocardial necrosis biomarkers. Elevations were defined as follows: creatine kinase that was more than twice the concentration of the upper limit of the normal range used by the local laboratory, a CK-MB fraction that was greater than the upper limit of the normal range, and a troponin I or T level that was more than twice the concentration of the upper limit of the normal range. “Short-term” was defined as the time period within 30 days of revascularization. Follow-up information was obtained by recording outcomes for patients via the telephone or in clinic.

### Statistical analysis

Continuous variables were presented as the mean  ±  standard deviation or median (with interquartile ranges). Categorical variables were expressed as percentages. Natural logarithmic transformation was used for continuous variables that had skewed distribution, including WBC, BUN, CR, TC, TG, LDL-C, HDL-C and FBG. Patients with 60 ≤ eGFR < 90 ml/(min · 1.73 m^2^) were identified as having mild renal dysfunction, and those with eGFR <60 ml/(min · 1.73 m^2^) were identified as having moderate or severe renal dysfunction. These cutoff values were selected on the basis of published guidelines^18^. Unpaired t test or Wilcoxon rank sum test was utilized to compare continuous variables between the PCI and CABG groups. Categorical variables were compared using Chi-square test or Fisher’s exact test. Breslow test was used to compare short-term outcomes between the PCI and CABG groups. Long-term outcomes were compared using log-rank test. The MACCE-free survival curve was estimated using the Kaplan-Meier method. Log-rank test was utilized to examine between-group differences in MACCE-free survival curves for categorical variables. Subgroup analyses were performed to evaluate the outcomes based on eGFR categories of 60–90 and <60 ml/(min · 1.73 m^2^).

This study used the univariate Cox proportional hazards model to identify potential predictors of MACCE. The proportional hazards assumption was visually assessed by using a Kaplan-Meier survival curve for categorical variables and was evaluated by testing the significance of the interaction between the variable and the time effect introduced into the Cox model for continuous variables. Variables with significant or borderline significant associations (P < 0.15) with outcomes and the interaction between non-proportional hazards variables and time effect were introduced into the stepwise time-dependent Cox model to evaluate the associations between factors and outcomes. The α for variable entering and retention in the multivariate model are 0.10 and 0.15, respectively. Subgroup analyses were performed to evaluate the associations between predictors and outcomes in subgroups based on the degree of renal dysfunction. All the time-dependent Cox models were adjusted for factors that are significant in the baseline characteristics analyses. All data were analyzed using the SAS 9.3 software.

## Results

### Baseline clinical and procedural characteristics

Between July 2003 and October 2005, 6005 coronary artery disease patients underwent revascularization at Beijing Anzhen Hospital. Among them, we identified 2923 ACS patients with renal dysfunction who underwent either PCI or CABG successfully after excluding patients with missing renal function measurements.

Baseline clinical and procedural characteristics are presented in Table 1. There were no significant differences between the PCI and CABG groups except for gender, hypertension, hemoglobin, BUN, HDL-C, OMI, ACS and the degree of renal dysfunction. There were 8.4% of ST-elevated MI patients in the CABG cohort and 22.8% in the PCI cohort. There were 3.6% of non-ST-elevated MI patients in the CABG cohort and 6.2% in the PCI cohort. 87.9% of patients had unstable angina in the CABG cohort and 71.1% in the PCI cohort. Chi-square test showed that the proportion of patients with ACS was different between two cohorts. Hemoglobin and HDL-C levels were lower in the CABG group, and BUN was higher in the CABG group. Males, OMI and moderate or severe renal dysfunction were more frequent in the CABG group than in the PCI group. Baseline procedural characteristics demonstrated that patients who underwent CABG tended to have multivessel disease, left main disease and left anterior descending disease. Complete revascularization was more frequent in the CABG group than in the PCI group.

**Table 1:** Baseline characteristics of ACS patients with renal dysfunction.

Variables	CABG (n = 1020)	PCI (n = 1903)	P value
Age	63.0 ± 9.2	62.4 ± 10.4	0.2173
Male	804(78.8)	1341(70.5)	<0.0001
BMI	25.9 ± 3.0	26.0 ± 3.2	0.6547
Hypertension	641(62.8)	1290(67.8)	0.0071
Diabetes mellitus	270(26.5)	504(26.5)	0.9935
Smoking history	395(38.7)	774(40.7)	0.3057
PVD	21(2.1)	41(2.2)	0.8641
Cerebrovascular disease history	103(10.1)	170(8.9)	0.3023
COPD	6(0.6)	20(1.0)	0.2041
Hemoglobin	134.7 ± 17.7	137.1 ± 16.4	0.0009
lnWBC	1.99 ± 0.32	1.98 ± 0.30	0.7794
Neutrophils	62.3 ± 12.3	62.5 ± 12.2	0.4624
lnBUN	2.83 ± 0.35	2.75 ± 0.33	<0.0001
lnLDL-C	4.59 ± 0.33	4.60 ± 0.30	0.2527
lnHDL-C	3.66 ± 0.22	3.68 ± 0.22	0.0213
lnTC	5.17 ± 0.24	5.18 ± 0.22	0.5115
lnTG	4.98 ± 0.52	4.96 ± 0.50	0.4436
lnFBG	4.65 ± 0.28	4.65 ± 0.27	0.2674
LVEF	60.0 ± 11.3	60.0 ± 11.2	0.843
OMI	325(31.9)	343(18.0)	<0.0001
ACS			<0.0001
STEMI	86(8.4)	433(22.8)	
NSTEMI	37(3.6)	117(6.2)	
UA	897(87.9)	1353(71.1)	
Atrial fibrillation and atrial flutter	19(1.9)	45(2.4)	0.3768
Moderate or severe (relative to mild) renal dysfunction	231(22.6)	364(19.1)	0.0243
Procedural characteristics			
Vessel involvement			<0.0001
1	83(8.1)	746(39.2)	
2	230(22.6)	656(34.5)	
3	707(69.3)	501(26.3)	
Left main disease	185(18.1)	79(4.2)	<0.0001
Ostial lesion	109(10.7)	163(8.6)	0.0599
Left anterior descending disease	501(49.1)	823(43.2)	0.0024
Complete revascularization	830(81.4)	1347(70.8)	<0.0001
Medications use			
β-blocker	969(95.0)	1661(87.3)	<0.0001
ACE-I	604(59.2)	1275(67.0)	<0.0001
ARB	57(5.6)	115(6.0)	0.6184
Calcium channel blocker	648(63.5)	593(31.2)	<0.0001
Statin	484(47.4)	1656(87.0)	<0.0001
Nitrate	964(95.4)	1557(82.8)	<0.0001

Mean  ±  standard deviation, or number of patients and percentage.

lnWBC, lnBUN, lnCR, lnLDL-C, lnHDL-C, lnTC, lnTG and lnFBG indicate the natural logarithmic transformation of WBC, BUN, CR, LDL-C, HDL-C, TC, TG and FBG, respectively.

### Short-term and long-term clinical outcomes

Short-term and long-term clinical outcomes after revascularization for ACS patients with renal dysfunction are shown in Table 2. Among these 2923 patients, 241 (8.24%) patients were lost to follow-up. The median follow-up time was 533 (441~661) days after revascularization. The incidence of short-term MACCE was 4.4% and 1.5% for the CABG and PCI cohorts, respectively. The incidence of long-term MACCE for ACS patients with renal dysfunction was 10.0% and 12.2% for CABG and PCI, respectively.

**Table 2:** Short-term and long-term clinical outcomes after revascularization.

Outcomes	Total			Mild Renal Dysfunction			Moderate or Severe Renal Dysfunction		
	CABG (n = 1020)	PCI (n = 1903)	P value	CABG (n = 789)	PCI (n = 1539)	P value	CABG (n = 231)	PCI (n = 364)	P value
Short-term outcomes									
All-cause mortality	29(2.8)	15(0.8)	<0.0001	14(1.8)	7(0.5)	0.0015	15(6.5)	8(2.2)	0.0090
Cardiac death	23(2.2)	14(0.7)	0.0005	13(1.6)	6(0.4)	0.0014	10(4.3)	8(2.2)	0.1437
MACCE	45(4.4)	28(1.5)	<0.0001	27(3.4)	18(1.2)	0.0002	18(7.8)	10(2.7)	0.0054
Long-term outcomes									
All-cause mortality	50(4.9)	51(2.7)	0.0017	26(3.3)	20(1.3)	0.0011	24(10.4)	31(8.5)	0.3783
Cardiac death	33(3.2)	35(1.8)	0.0168	19(2.4)	12(0.8)	0.0012	14(6.1)	23(6.3)	0.9822
MACCE	102(10.0)	232(12.2)	0.1124	68(8.6)	173(11.2)	0.0638	34(14.7)	59(16.2)	0.7897

Number of patients and percentage.

P value for short-term outcome was calculated using Breslow test. P value for long-term outcome was calculated by log-rank test.

**Figure 1:**
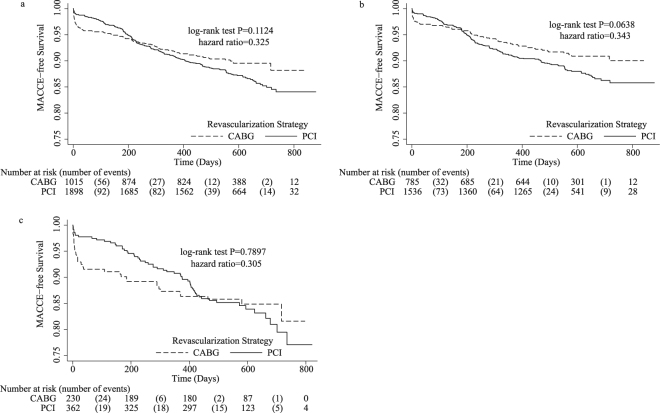
MACCE-free Kaplan-Meier survival curve. (**a**) MACCE-free Kaplan-Meier survival curve for ACS patients with renal dysfunction undergoing CABG versus PCI. (**b**) MACCE-free Kaplan-Meier survival curve for the mild renal dysfunction subgroup undergoing CABG versus PCI. (**c**) MACCE-free Kaplan-Meier survival curve for the moderate or severe renal dysfunction subgroup undergoing CABG versus PCI. Hazard ratios represent the adjusted hazard ratios of PCI relative to CABG at the initial time after revascularization. ACS, Acute coronary syndrome; CABG, Coronary Artery Bypass Graft; MACCE, major adverse cardiac and cerebrovascular events; PCI, Percutaneous Coronary Intervention.

Figure 1a shows the MACCE-free Kaplan-Meier survival curves for ACS patients with renal dysfunction after CABG or PCI. Two MACCE-free Kaplan-Meier survival curves were crossed in Fig. 1a. Breslow test showed that the short-term MACCE-free survival rate difference between the PCI and CABG cohorts was significant (χ^2^ = 22.86, P < 0.0001). Log-rank test showed that the long-term MACCE-free survival rate difference between the two cohorts was insignificant (χ^2^ = 2.52, P = 0.1124).

Subgroup analyses showed that the incidence of short-term MACCE was lower in the PCI cohort than that in the CABG cohort in both the mild renal dysfunction (1.2% versus 3.4%, χ^2^ = 13.63, P = 0.0002) and the moderate or severe renal dysfunction (2.7% versus 7.8%, χ^2^ = 7.73, P = 0.0054) subgroups. Log-rank test showed that the incidence of long-term MACCE was insignificantly different between the PCI and CABG cohorts in both the mild renal dysfunction (11.2% versus 8.6%, χ^2^ = 3.44, P = 0.0638) and the moderate or severe renal dysfunction (16.2% versus 14.7%, χ^2^ = 0.071, P = 0.7897) subgroups. The PCI and CABG MACCE-free survival rate Kaplan-Meier curves crossed in both subgroups (Fig. 1b and c). The MACCE-free survival rate in the moderate or severe renal dysfunction subgroup appeared to fall faster than that in the mild renal dysfunction subgroup.

### Predictors of clinical outcomes in ACS patients with renal dysfunction undergoing revascularization

Predictors of clinical outcomes in ACS patients with renal dysfunction were identified using the univariate Cox model and the stepwise time-dependent Cox model. The univariate analysis identified age, LVEF, hemoglobin, neutrophils, BUN, vessel involvement, diabetes mellitus, hypertension, revascularization strategy, complete revascularization, renal dysfunction degree, WBC, FBG, left main disease, left anterior descending disease, the type of ACS, and use of statin, ACE-I, ARB, calcium channel blocker and nitrate as potential predictors of MACCE. All predictors identified by univariate analysis were introduced into a stepwise time-dependent Cox regression model. Subgroup analyses were performed to assess the associations between these predictors and their outcomes in subgroups based on the degree of renal dysfunction by using a time-dependent Cox regression model. All the time-dependent Cox models were adjusted for factors that are significant in the baseline characteristics analyses. The results are shown in Table 3.

**Table 3:** The adjusted time-dependent Cox models for predictors of MACCE.

variables	Total			Mild Renal Dysfunction			Moderate or Severe Renal Dysfunction		
	Adjusted HR	95% CI	P value	Adjusted HR	95% CI	P value	Adjusted HR	95% CI	P value
**Vessel involvement**									
1-vessel (relative to 3-vessel)	0.625	0.439–0.889	0.0090	0.626	0.415–0.943	0.0250	0.605	0.296–1.233	0.1663
2-vessel (relative to 3-vessel)	0.758	0.567–1.012	0.0602	0.762	0.540–1.076	0.1224	0.682	0.393–1.185	0.1749
ARB	1.887	1.276–2.792	0.0015	1.950	1.220–3.117	0.0052	1.904	0.898–4.036	0.0929
PCI (relative to CABG)	0.325	0.162–0.652	0.0015	0.343	0.147–0.802	0.0135	0.305	0.089–1.043	0.0584
PCI (relative to CABG) × ln(time + 1)	1.411	1.230–1.619	<0.0001	1.372	1.163–1.619	0.0002	1.485	1.158–1.904	0.0018
Moderate or severe (relative to mild) renal dysfunction	1.335	0.996–1.788	0.0530	—	—	—	—	—	—
Left main disease	1.425	0.980–2.072	0.0635	1.211	0.743–1.973	0.4420	1.929	1.023–3.634	0.0422
LVEF	0.960	0.936–0.985	0.0021	0.973	0.941–1.007	0.1166	0.945	0.906–0.986	0.0094
LVEF × ln(time + 1)	1.005	1.000–1.011	0.0449	1.003	0.996–1.010	0.3513	1.008	0.999–1.016	0.0933
Diabetes mellitus × ln(time + 1)	1.063	1.011–1.118	0.0175	1.037	0.975–1.103	0.2525	1.110	1.008–1.222	0.0339
ACE-I × ln(time + 1)	1.033	0.978–1.091	0.2414	1.055	0.989–1.125	0.1037	0.983	0.885–1.092	0.7531

predictor × ln(time + 1) indicates the interaction between the predictor and time effect (natural logarithmic transformation of ‘time + 1’).

The stepwise time-dependent Cox model identified LVEF, vessel involvement, ARB, revascularization strategies, the degree of renal dysfunction and diabetes mellitus as predictors for MACCE. Among them, revascularization strategies and diabetes mellitus were identified as time-dependent covariates. Then, the time-dependent Cox model was adjusted for unbalanced baseline factors between the PCI and CABG groups. Our research indicated that the regression coefficient of revascularization strategies is ‘−1.124 + 0.344 × ln (time + 1)’. In algebra, PCI was superior to CABG in ACS patients with renal dysfunction within approximately 25 days of revascularization. After this period, CABG had an advantage over PCI in this high-risk group. When compared with ACS patients with renal dysfunction receiving CABG, those undergoing PCI were associated with a lower hazard for MACCE (adjusted HR, 0.325; 95% confidence interval, 0.162–0.652; χ^2^ = 10.02, P = 0.0015 < 0.01) initially after revascularization. The interaction between PCI and time effect was associated with an increased hazard relative to CABG for MACCE (adjusted HR, 1.411; 95% confidence interval, 1.230–1.619; χ^2^ = 24.13, P < 0.01). Additionally, the results indicated that the interaction between diabetes mellitus and time effect was hazardous for MACCE (adjusted HR, 1.063; 95% confidence interval, 1.011–1.118; χ^2^ = 5.65, P = 0.0175).

Subgroup analyses indicated that the hazard ratio of PCI relative to CABG was time-dependent in both the mild renal dysfunction and the moderate or severe renal dysfunction subgroups. In the mild renal dysfunction subgroup, the regression coefficient of the revascularization strategy was ‘−1.071 + 0.316 × ln (time + 1)’. At the initial time after revascularization, PCI was associated with a lower hazard for MACCE (adjusted HR, 0.343; 95% confidence interval, 0.147–0.802; χ^2^ = 6.10, P = 0.0135) in this subgroup. The interaction between PCI and time effect was associated with an increased hazard ratio relative to CABG for MACCE (adjusted HR, 1.372; 95% confidence interval, 1.163–1.619; χ^2^ = 14.04, P = 0.0002). In the moderate or severe renal dysfunction subgroup, the regression coefficient of the revascularization strategy was ‘−1.188 + 0.395 × ln (time + 1)’. In this subgroup, PCI was associated with a borderline significant lower hazard for MACCE (adjusted HR, 0.305; 95% confidence interval, 0.089–1.043; χ^2^ = 3.58, P = 0.0584) at the initial time after revascularization. The interaction between PCI and time effect was associated with an increased hazard ratio relative to CABG for MACCE (adjusted HR, 1.485; 95% confidence interval, 1.158–1.904; χ^2^ = 9.73, P = 0.0018). Additionally, subgroup analyses showed that diabetes mellitus was a time-dependent covariate only in the moderate or severe renal dysfunction subgroup.

## Discussion

ACS patients with renal dysfunction typically have worse outcomes than those with normal renal function. Renal dysfunction is an independent risk factor for intraoperative and postoperative adverse events^19^. Impaired renal function may be associated with increased inflammation and oxidative stress. It may also be associated with certain physical changes including high levels of homocysteine, hyperuricemia, hypercalcemia and uremia, which may lead to a poor prognosis^2,20^. Additionally, patients with renal dysfunction tend to be of advanced age and have hypertension and diabetes mellitus. These concomitant factors may increase MACCE risk. Patients with renal dysfunction tend to have a greater frequency of multivessel disease and left main disease^21^. The adverse consequences of renal dysfunction may reduce oxygen supply to the myocardium due to severe damage to the epicardial coronary macrovessels and depressed coronary reserve secondary to microvessel disease^22^. PCI and CABG are alternative revascularization strategies for ACS patients with renal dysfunction. However, there is a lack of research assessing a time-dependent relative risk of PCI relative to CABG in this high-risk group. Which revascularization strategy is optimal in different situations is still controversial. Consequently, evaluating the fluctuant relative risk of revascularization strategies by applying scientific methods in this study is crucial for surgical decision making.

This study used MACCE as outcomes due to the small incidence of separate events. Our results showed that the cumulative incidence of short-term MACCE was higher after CABG when compared with PCI in ACS patients with renal dysfunction. Subgroup analyses showed similar results. These findings may be because severe infection, major bleeding, stroke, respiratory dysfunction and other non-cardiac complications were more frequently seen in the CABG cohort than in the PCI cohort^23–25^. Simultaneously, PCI had lower adverse effects on other organs or systems, that may explain the lower incidence of short-term MACCE in the PCI cohort relative to the CABG cohort^26^. However, the results indicated that long-term MACCE rates were not significantly different between the two revascularization strategies in this high-risk group, a finding that may differ from other studies in the literature. Additionally, subgroup analyses also yielded similar results. The SYNTAX trial demonstrated that, in patients with renal dysfunction, CABG was superior to PCI for a lower incidence of MACCE (15.6% vs. 27.4%, P < 0.05)^27^. Aoki J. *et al*. analyzed the data from the ARTS trial and found that 5-year MACCE rates were lower after CABG when compared with PCI (32% vs. 49%, P < 0.05) in multivessel coronary artery disease patients with renal dysfunction^28^. An observational study reported similar conclusions from the data from a large Northern California registry^29^. Another observational study indicated that the CABG cohort had greater 1-year, 2-year and 3-year freedom from MACCE rates (89.4% vs. 71.2%, 81.9% vs. 60.5%, 75.2% vs. 51.8%, respectively, P < 0.05) than the PCI cohort in multivessel disease patients with chronic kidney disease^30^. The results from the paper are different from those in the literature, because the risk of MACCE after revascularization was not proportional (Fig. 1) for the entire object group and separate subgroups. Specifically, the overall differences in MACCE-free survival rates between the two revascularization cohorts is insignificant, because an early disadvantage of CABG relative to PCI for MACCE reduces the entire advantage of CABG for MACCE when compared with PCI, as shown in Fig. 1.

A few studies have used the Cox proportional hazardous model, the stratified or the weighted Cox model to assess the hazard ratio of PCI relative to CABG for MACCE. However, the hazard ratio of revascularization strategies is time-dependent. A proportional hazardous assumption may not be met. Consequently, typical models may not assess the time-dependent hazard ratio. To evaluate the fluctuant relative effect of two revascularization strategies on MACCE in ACS patients with renal dysfunction, the paper applied a time-dependent Cox model. Time-dependent Cox model characteristics may introduce time-dependent covariates into the model to assess their fluctuant associations with outcomes. The paper indicated that LVEF, vessel involvement, ARB, revascularization strategies, the degree of renal dysfunction and diabetes mellitus were risk factors for MACCE. Among them, revascularization strategies and diabetes mellitus were identified as time-dependent covariates. The study indicated that the regression coefficient of revascularization strategies was ‘−1.124 + 0.344 × ln (time + 1)’ in the entire object group. In algebra, PCI was superior to CABG in ACS patients with renal dysfunction within approximately 25 days of revascularization. After this period, ACS patients with renal dysfunction receiving CABG had a lower risk for MACCE in this high-risk group when compared to those receiving PCI. Initially, there is a high rate of restenosis after PCI surgery. Repeat revascularization may be more frequently required for patients undergoing PCI than CABG. By contrast, CABG provides prophylactic protection against future coronary events by passing vulnerable plaques that are typically located in the proximal coronary tree^31^. Additionally, Table 1 indicated that complete revascularization was more frequent in the CABG cohort than in the PCI cohort. Certain studies have suggested that complete revascularization has a survival advantage over incomplete revascularization^32^. Similar regularity was found in the subgroup analyses. In addition, subgroup analyses indicated that, at the initial time after revascularization, the adjusted hazard ratio of PCI relative to CABG appeared to be lower in the moderate or severe renal dysfunction subgroup than that in the mild renal dysfunction subgroup (0.305 versus 0.343, respectively). However, the adjusted hazard ratio of PCI relative to CABG in the moderate or severe renal dysfunction subgroup appeared to rise faster with time than that in the mild renal dysfunction subgroup. In addition, subgroup analyses indicated that diabetes mellitus, a traditional risk factor for MACCE in ACS patients, was identified as a time-dependent covariate in the moderate or severe renal dysfunction subgroup, but not in the mild renal dysfunction subgroup. These findings indicated that diabetes mellitus has an increased risk for MACCE with time in the moderate or severe renal dysfunction patients that may provide useful information for clinical practice.

Our study has a few limitations. The study used data from a retrospective registry. Selection bias may occur due to a few baseline characteristics variables that were not balanced between the two groups. Approximately 40 variables were initially included in the present study. However, a few unremarked factors may have an effect on MACCE in ACS patients with renal dysfunction. These unmeasured confounders may generate a certain bias when evaluating associations between major factors and outcomes. Figure 1 and log rank test indicated that there were no significant difference for the MACCE-free survival rate between the two revascularization strategies. This finding may be due to disproportion of MACCE risk after revascularization and insufficient follow-up time. Perhaps significant MACCE-free survival rates difference between PCI and CABG may appear if follow-up time were extended. Additionally, our study utilized a time-dependent Cox model to assess the fluctuant associations between risk factors and MACCE. Values of these time-dependent covariates remained unchanged in the present study. A few variables, especially laboratory variables, may vary with time. However, these fluctuant data were not recorded in the present study. Consequently, the Cox mixed effect model may not be applied in our study to evaluate individual risk factors associations with outcomes.

In conclusion, for the entire object group and separate subgroups based on the degree of renal dysfunction, the revascularization strategy was identified as a time-dependent covariate for MACCE. PCI tends to have a lower risk for MACCE when compared with CABG in the short term. However, the hazard ratio for PCI relative to CABG increases with time.
